# Whether to report diabetes as the underlying cause-of-death? a survey of internists of different sub-specialties

**DOI:** 10.1186/1472-6823-10-13

**Published:** 2010-07-23

**Authors:** Tsung-Hsueh Lu, Ching-Fai Kwok, Low-Tone Ho

**Affiliations:** 1 Institute of Public Health, College of Medicine, National Cheng Kung University, Tainan, Taiwan; 2 Division of Endocrinology and Metabolism, Department of Medicine and Department of Medical Research and Education, Taipei Veterans General Hospital, Taipei, Taiwan; 3 Faculty of Medicine and Institutes of Physiology and Clinical Medicine, School of Medicine, National Yang Ming University, Taipei, Taiwan

## Abstract

**Background:**

Cause-specific mortality is a commonly used endpoint of clinical trials or prospective studies. However, it is sometimes difficult for physician to determine the underlying-cause-of-death (UCD), especially for diabetic patients coexisted with cardiovascular diseases (CVD). The aim of this survey was to examine whether internists with different specialties have different opinions on the reporting of diabetes as the UCD.

**Methods:**

A total of 549 physicians completed the questionnaire in Taiwan, which comprised seven hypothetical case scenarios, each indicating a different level of contribution of diabetes in initiating the chain of events leading to death.

**Results:**

As a whole, endocrinologists were more likely than cardiologists and nephrologists to report diabetes as the UCD. The differences were more prominent when the diabetic patient had a coexisting CVD. In scenario 3 (a diabetic patient with hypertension who died from acute myocardial infarction), the percentage was 56% in endocrinologists, which was significantly higher than in cardiologists (42%) and nephrologists (41%). In scenario 4 (a diabetic patient with hypertension who died from cerebrovascular infarction), the percentage was 45% in endocrinologists, and only 31% in cardiologists and 36% in nephrologists.

**Conclusions:**

Internists of different sub-specialties do have different opinions on the reporting of diabetes as the UCD, especially when the diabetic patient has a coexisting CVD.

## Background

Cause-specific mortality is a commonly used endpoint of clinical trials or prospective studies. Cause-of-death data are tabulated according to the underlying cause-of-death (UCD), which is defined by the World Health Organization as "the disease or injury which initiated the train of morbid events leading directly to death" [1]. One of the difficulties faced by physicians in completing the cause-of-death certification is to decide which disease "initiated" this train. Given the same clinical case scenarios of a patient, different physicians might identify different diseases as having "initiated" the train. For example:

Sepsis → Death

Pneumonia → Sepsis → Death

Stroke → Pneumonia → Sepsis → Death

Coronary heart disease → Stroke → Pneumonia → Sepsis → Death

Diabetes mellitus → Coronary heart disease → Stroke → Pneumonia → Sepsis → Death

All of the above statements are in correct causal sequence and are acceptable; however, the UCD assigned for each statement is different. The cause-of-death section of the death certificate is designed to elicit the opinion of the medical certifier, and the reported cause of death represents a medical opinion that might vary among individual physicians [2].

When a person with diagnosed diabetes dies, different physicians might have different opinions on whether the death process was "initiated" by the diabetes. Studies using case scenarios with diabetes have indicated that physicians show great variations in the reporting of diabetes as the UCD [3-5]. Despite the existence of many studies that have investigated the factors associated with the reporting of diabetes on death certificates [6-14], few have examined the possible effects of the characteristics of certifiers [10-14]. Two studies have suggested that primary physicians are more likely to report diabetes on death certificates [10,14]. Nonetheless, little is known about whether internists of different sub-specialties have differing opinions on the reporting of diabetes as the UCD.

The aim of this study was therefore to examine whether endocrinologists, cardiologists, and nephrologists have different opinions on the reporting of diabetes as the UCD. The rationale for choosing the aforementioned sub-specialties is several-fold. Most diabetic patients are cared for by internists of different sub-specialties: the presence of endocrinologists is obvious due to their role in the diagnosis and management of diabetes; the other two sub-specialties are involved in the outcomes and complications of diabetes, with cardiologists present for the macro-angiopathy of diabetes and nephrologists for the micro-angiopathy of diabetes.

## Methods

### Scope of this study

The process of production of the UCD for official mortality tabulation consist two steps: 1) certification by physicians and 2) coding according to the ICD rules. This study dealt only with the certification process. Previous studies using diabetes-related case scenarios to examine physicians' certification behavior asked the physicians to complete the cause-of-death section on the dummy death certificate for each case scenario [3-5]. We did not use dummy death certificate in this study because many physicians reported incorrect causal sequences [4,15], which could not provide useful information in judging relative role of diabetes in contributing to death. We thus listed all possible correct layouts of each scenario and let the physician choosing the most suitable layout. Furthermore, we did not provide detail clinical information like previous studies did [3-5] we thus could not provide a reference UCD in each scenario.

### Participants

This study has been approved by National Cheng Kung University Institutional Review Board. With the help of the Diabetes Association, the Society of Cardiology and the Society of Nephrology in Taiwan, questionnaires were mailed to members with sub-specialty qualifications, and three waves of reminders were sent to those who did not return the questionnaire. A total of 549 physicians returned the questionnaire, representing a response rate of 26% (549/2076). The response rate varied with sub-specialty, and was 34% (116/340) for endocrinologists, 19% (190/1000) for cardiologists, and 33% (243/736) for nephrologists.

### Survey questionnaire

**Figure 1:**
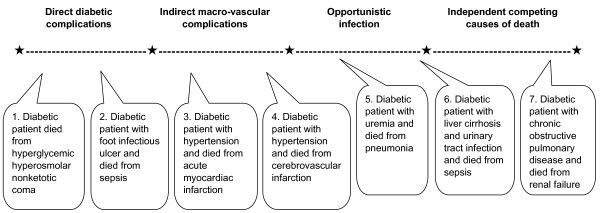
**The seven hypothetical case scenarios indicating different level of contribution of diabetes in initiating the chain of events leading to death**.

The questionnaire comprised seven hypothetical case scenarios, each indicating a different level of contribution of diabetes in initiating the chain of events leading to death. Some of the case scenarios were modified from the study by Balkau *et al. *[3]. In scenarios 1 and 2, the diabetic patient died from more 'direct' diabetic complications, i.e., hyperglycemic hyperosmolar nonketotic coma and sepsis due to a diabetic foot ulcer. In scenarios 3 and 4, the diabetic patient died from more 'indirect' macro-vascular complications, such as acute myocardial infarction (AMI) and cerebrovascular infarction. In scenarios 5 and 6, the diabetic patient died from 'opportunistic' infection, e.g., pneumonia and urinary tract infection. In scenario 7, the diabetic patient died from 'independent' competing causes of death, e.g., respiratory failure owing to chronic obstructive pulmonary disease. We hypothesized that the percentage of cases in which diabetes was reported as the UCD would decrease from scenario 1 through 7, because the role that diabetes played in "initiating" the death process decreased from scenario 1 to 7 (Figure 1).

To avoid the responding physician reporting incorrect causal sequence on dummy death certificate for each case scenario, we firstly provided an instruction on cause-of-death certification published by the Department of Health of Taiwan was included along with the questionnaire to remind the physicians how to correctly certify cause-of-death statements. We then used a specific choice form for each case scenario, which listed all possible 'correct' layouts of cause-of-death statements. Thus, the chosen causal relationship between diseases and the assigned UCD represented the real intent of the certifying physician. Please see the questionnaire [Additional file 1].

### Statistical analysis

The main outcome of this study was the percentage of cases in which diabetes was reported as the UCD by the participating physicians. We used 95% confidence intervals (95% CI) to examine the differences in the percentages for various hypothetical case scenarios, and further compared the differences in percentages by sub-specialty using the chi-square test.

## Results

The characteristics of the responding physicians are shown in Table 1. The age distribution of cardiologists was a little bit different from the other two specialists, which composed more aged respondents. A higher percentage of nephrologists practiced in clinics (most of these clinics were dialysis centers). Only half of the responding physicians had issued a death certificate within the past half-year.

**Table 1:** Characteristics of respondents by sub-specialty

	Endocrinologists		Cardiologists		Nephrologists	
			
	*n*	%	*n*	%	*n*	%
**Total**	116	100.0	190	100.0	243	100.0
**Age (years)**						
≤ 39	31	26.7	43	22.6	64	26.3
40-49	55	47.4	69	36.3	124	51.0
50-59	25	21.6	51	26.9	49	20.2
≥ 60	5	4.3	27	14.2	6	2.5
**Practice type**						
Medical center	43	37.1	73	38.4	46	19.1
Regional hospital	45	38.8	62	32.6	48	19.9
Area hospital	16	13.8	22	11.6	60	24.9
Clinic	12	10.3	33	17.4	87	36.1

The percentage of cases in which diabetes was reported as the UCD differed greatly across case scenarios, raging from 99.6% for scenario 1 to 1.8% for scenario 7 (Table 2). Generally, the ranking of percentages by scenario followed the order we hypothesized; one exception, though not statistically significant, was the percentage for scenario 6, which was higher than we expected. In other words, physicians thought that diabetes played a more significant role in "initiating" opportunistic infections than in "initiating" cerebrovascular diseases.

**Table 2:** Percentage (%) of respondents reporting diabetes as the underlying cause of death in various hypothetical case scenarios by sub-specialty

	All respondents		Endocrinologists		Cardiologists		Nephrologists	
				
Hypothetical case scenarios	%	(No)	%	(No)	%	(No)	%	(No)
1. Diabetic patient died from hyperglycemic hyperosmolar nonketotic coma	100	(542/544)	100	(114/114)	99	(186/188)	99	(237/240)
2. Diabetic patient with foot infectious ulcer and died from sepsis	78	(422/543)	78	(87/112)	76	(144/189)	79	(191/242)
3. Diabetic patient with hypertension and died from acute myocardiac infarction	44	(239/539)	56	(62/110)	41	(78/188)	41	(99/241)
4. Diabetic patient with hypertension and died from cerebrovascular infarction	36	(194/541)	45	(49/110)	31	(59/189)	36	(86/242)
5. Diabetic patient with uremia and died from pneumonia	38	(206/542)	43	(49/110)	38	(71/187)	36	(86/241)
6. Diabetic patient with liver cirrhosis and urinary tract infection and died from sepsis	42	(228/540)	45	(50/111)	39	(74/189)	43	(104/240)
7. Diabetic patient with chronic obstructive pulmonary disease and died from renal failure	2	(10/545)	2	(2/114)	3	(6/189)	1	(2/242)

Overall, a higher percentage of endocrinologists reported diabetes as the UCD as compared with cardiologists and nephrologists (Table 2). The differences were more prominent when the diabetic patient died from a macro-vascular disease. In scenario 3 (a diabetic patient with hypertension who died from AMI), the percentage was 56% in endocrinologists, which was significantly higher than in cardiologists (42%) and nephrologists (41%). In scenario 4 (a diabetic patient with hypertension who died from cerebrovascular infarction), the percentage was 45% in endocrinologists, 31% in cardiologists and 36% in nephrologists. For the other scenarios, no significant differences between sub-specialists were found.

## Discussion

Our results suggest that internists of different sub-specialties have different opinions on the reporting of diabetes as the UCD with regard to the role that diabetes played in "initiating" the chain of events leading to death. We also found that, at least in Taiwan, endocrinologists are more likely than cardiologists and nephrologists to report diabetes as the UCD, especially when diabetic patients die from macro-vascular complications such as AMI and cerebrovascular infarction.

One strength of this study was that we simplified the case scenarios and standardized the wordings, explicitly asking the certifying physicians whether diabetes "initiated" the death process, which could reduce the diversity in interpreting the same case scenarios. The second strength was the use of choice form which listed all correct layouts for each case scenario thus could avoid the reporting of incorrect causal sequences by responding physicians.

One of the limitations of this study was that the response rate 26% (549/2076) in this study was not very satisfactory compared with previous studies, which ranged from 56% (168/300) to 91% (274/300) in a European study [3], 86% (124/145) among general practitioners in a Taiwan study [4] and 12% (590/4800) among residents in an US study [5]. However, we don't think there was possible reason to assume that characteristics of non-respondent in particular specialist differed greatly from other specialists (i.e., misclassification bias) and will bias our conclusions. The second limitation was that, owing to the resources available, we compared only three sub-specialties. In future studies we could include internists of other sub-specialties, such as infectious diseases, or compare the results among residents.

Some of the case scenarios in this study were modified from the study by Balkau *et al. *[3]; we can therefore make robust comparisons between physicians in Taiwan and physicians in European countries. For patients who die from a diabetic coma, physicians of the different countries unanimously agree that diabetes should be recorded as the UCD. For diabetic patients who die from AMI, the percentage of cases in which diabetes is reported as the UCD ranged from 3% in France to 26% in Northern Ireland, both of which are much lower than in Taiwan (44%). By the same token, for diabetic patients who die from cerebrovascular disease, the percentage was found to be less than 15% in European countries, but was 36% in Taiwan.

One of the possible reasons why Taiwanese physicians reported higher percentage in selecting diabetes as the UCD was that the status of diabetes management in Taiwan was suboptimal. According to a cohort study of 2446 patients (from 25 diabetic centers) with more than 12 months of diabetes management found that 59% of participants had HbA1c >7.4% [16]. Another nationwide surveys to evaluate the status of diabetes control in 7541 diabetes subjects among 114 accredited Diabetes Health Promotion Centers in Taiwan in 2006 indicated that only 32.4% of subjects whose HbA1c levels was less than 7% [17]. The authors of above mentioned two studies all concluded that the majority of Taiwanese patients had unsatisfactory glycaemic control which may lead to diabetes complications.

Another indirect evidence was that given similar percentage of reporting diabetes on death certificate between Taiwan, Australia and Sweden, Taiwanese physicians were more likely than their counterpart physicians in Australia and Sweden to report diabetes on part I of death certificates, which results in a higher percentage of cases in which diabetes is assigned as the UCD [12]. Little is known as to whether the high percentage of cases in which diabetes is reported on part I of the death certificate is due to the real intent of certifying physicians or to errors in cause-of-death certification. According to the results of this study, we could conclude that the high percentage of cases in which diabetes is reported on part I of the death certificate was a reflection of the real intent of Taiwanese physicians.

In terms of the high percentage of reporting diabetes as the UCD by Taiwanese physicians, especially when the diabetic patients coexisted with AMI and cerebrovascular diseases, we suggest the following recommendations. As the cause-of-death section of death certificate is designed according to preventive medicine, the certifying physicians could evaluate how well the prevention has been done. If diabetes were well control (e.g., HbA1c lower than 7%), we suggest that certifiers could enter diabetes in Part II of the death certificate. On the contrary, if the patient did not control blood sugar level well and it is highly possible that diabetes was the perpetrator of cardiovascular diseases, then the certifiers could enter diabetes in Part I of the death certificate.

## Conclusions

Despite the low response rates, the findings of this study concord with the original hypothesis about the hierarchy in the percentages of case scenarios for which diabetes is reported as the UCD, i.e., from 'direct' diabetic complications to 'indirect' macro-vascular complications to 'opportunistic' infection and then 'independent' competing causes of death. This study further suggests that internists of different sub-specialties have different opinions on the reporting of diabetes as the UCD, especially when the diabetic patient has a coexisting cardiovascular disease. Because of these certifier preferences, underlying cause statistics are not entirely reliable. Therefore, in addition to standardizing certification practices, the authors could also advocate more research using multiple causes of death, which include analyses that use information from Part II of the death certificate.

## Competing interests

The authors declare that they have no competing interests.

## Authors' contributions

THL initiated the idea and conducted the primary data analyses. THL, CFK, LTH and SW equally participated in the interpretation of the results, critically commented and drafted the manuscript. All the authors have read and approved the final version of manuscript.

## Acknowledgements

This study was supported by a grant from the National Science Council of Taiwan (NSC92-2320-B-040-039) and the Department of Health of Taiwan (DOH96-TD-M113-049 and 99Z4001)

## Pre-publication history

The pre-publication history for this paper can be accessed here:

http://www.biomedcentral.com/1472-6823/10/13/prepub

## Supplementary Material

Additional file 1 **Questionnaire of Diabetes-Related Cause-of-Death Certification**. The questionnaire used in this study. Click here for file
