# Supplementary material for: Whether to report diabetes as the underlying cause-of-death? a survey of internists of different sub-specialties
Source: BMC Endocr Disord. 2010 Jul 23;10:13. doi: 10.1186/1472-6823-10-13 (PMC2912904; doi:10.1186/1472-6823-10-13)
Supplement: Additional file 1 — Questionnaire of Diabetes-Related Cause-of-Death Certification. The questionnaire used in this study. [file 1472-6823-10-13-S1.PDF]

Questionnaire of Diabetes-Related Cause-of-Death Certification

Diabetes is an important risk factor of cardiovascular disease and infection, which made physicians difficult in certifying cause-of-death on death certificate. This study was supported by the National Science Council of Taiwan and aimed to understand how physician certifying diabetes-related cause-of-death. Please spend some time in filling the questionnaire. Your information is very important for department of health to design a better instruction for teaching physicians how to correctly reporting the cause-of-death on death certificate. If you have any questions in filling the questionnaire, please do not hesitate to call Dr. Lu (0928389971) of National Cheng University, College of Medicine, Institute of Public Health, Tainan701, Taiwan.

Basic information

Age:______years old; Sub-specialty:__________。

Facility: ☐ Medical Center；☐ Regional hospital；☐ Areal hospital；☐ Clinics

Have you issued death certificate recent half year? ☐ No；☐ Yes，How many?：______ 。

1. If the deceased patient you cared had long history of diabetes and hypertension and finally died from acute cardiac infarction (AMI), how would you report the cause-of-death for this patient? (Please select one of the following possible reporting forms)

☐ Part I	☐ Part I	☐ Part I	☐ Part I
a) AMI	a) AMI	a) AMI	a) AMI
b) Hypertension	b) Diabetes	b) Hypertension	b)
c) Diabetes	c)	c)	c)
Part II：	Part II：Hypertension	Part II：Diabetes	Part II：Diabetes, Hypertension

2. If the deceased patient you cared had long history of diabetes and hypertension and finally died from cerebral infarction (CI), how would you report the cause-of-death for this patient? (Please select one of the following possible reporting forms)

☐ Part I	☐ Part I	☐ Part I	☐ Part I
a) CI	a) CI	a) CI	a) CI
b) Hypertension	b) Diabetes	b) Hypertension	b)
c) Diabetes	c)	c)	c)
Part II：	Part II：Hypertension	Part II：Diabetes	Part II：Diabetes, Hypertension

3. If the deceased patient you cared had long history of diabetes and had foot infectious ulcer and finally died from sepsis, how would you report the cause-of-death for this patient? (Please select one of the following possible reporting forms)

□ Part I	□ Part I	□ Part I
a) Sepsis	a) Sepsis	a) Sepsis
b) Foot infectious ulcer	b) Foot infectious ulcer	b)
c) Diabetes	c)	c) Diabetes
Part II：	Part II：Diabetes	Part II：Diabetes, Foot infectious ulcer

4. If the deceased patient you cared had long history of diabetes and liver cirrhosis and had urinary tract infection (UTI) and finally died from sepsis, how would you report the cause-of-death for this patient? (Please select one of the following possible reporting forms)

□ Part I	□ Part I	□ Part I
a) Sepsis	a) Sepsis	a) Sepsis
b) UTI	b) UTI	b) UTI
c) Diabetes	c) Liver cirrhosis	c)
Part II：Liver cirrhosis	Part II：Diabetes	Part II：Diabetes, Liver cirrhosis

5. If the deceased patient you cared had long history of diabetes and receiving hemodialysis because of chronic renal failure (CRF) and finally died from pneumonia, how would you report the cause-of-death for this patient? (Please select one of the following possible reporting forms)

□ Part I	□ Part I	□ Part I
a) Pneumonia	a) Pneumonia	a) Pneumonia
b) CRF	b) CRF	b)
c) Diabetes	c)	c)
Part II：	Part II：Diabetes	Part II：Diabetes, CRF

6. If the deceased patient you cared had long history of diabetes and chronic obstructive pulmonary disease (COPD) and finally died from respiratory failure, how would you report the cause-of-death for this patient? (Please select one of the following possible reporting forms)

□ Part I	□ Part I	□ Part I
a) Respiratory failure	a) Respiratory failure	a) Respiratory failure
b) Diabetes	b) COPD	b)
c)	c)	c)
Part II：COPD	Part II：Diabetes	Part II：Diabetes, COPD
